# Low prevalence of influenza A strains with resistance markers in Brazil during 2017–2019 seasons

**DOI:** 10.3389/fpubh.2022.944277

**Published:** 2022-09-14

**Authors:** Thiago das Chagas Sousa, Jessica Santa Cruz Carvalho Martins, Milene Dias Miranda, Cristiana Couto Garcia, Paola Cristina Resende, Cliomar A. Santos, Maria do Carmo Debur, Rodrigo Ribeiro Rodrigues, Andrea Cony Cavalcanti, Tatiana Schäffer Gregianini, Felipe Campos de Melo Iani, Felicidade Mota Pereira, Sandra Bianchini Fernandes, Jessylene de Almeida Ferreira, Katia Correa de Oliveira Santos, Fernando Motta, David Brown, Walquiria Aparecida Ferreira de Almeida, Marilda Mendonça Siqueira, Aline da Rocha Matos

**Affiliations:** ^1^Laboratory of Respiratory Viruses and Measles, Oswaldo Cruz Institute, FIOCRUZ Fundation, Rio de Janeiro, Brazil; ^2^Laboratório Central de Saúde Publica de Sergipe (LACEN-SE), Aracaju, Sergipe, Brazil; ^3^Laboratório Central do Estado do Paraná (LACEN-PR), Curitiba, Paraná, Brazil; ^4^Laboratório de Saúde Pública do Estado do Espírito Santo, Secretaria de Saúde do Estado do Espírito Santo (LACEN-ES), Vitória, Espirito Santo, Brazil; ^5^Núcleo de Doenças Infecciosas, Universidade Federal do Espírito Santo, Vitória, Espirito Santo, Brazil; ^6^Laboratório Central de Saúde Pública do Rio de Janeiro (LACEN-RJ), Rio de Janeiro, Brazil; ^7^Laboratório Central de Saúde Pública da Secretaria de Saúde do estado do Rio Grande do Sul, (LACEN-RS)/CEVS/SES-RS, Porto Alegre, Rio Grande do Sul, Brazil; ^8^Laboratório Central de Saúde Pública de Minas Gerais (LACEN-MG), Fundação Ezequiel Dias, Belo Horizonte, Minas Gerais, Brazil; ^9^Laboratório Central da Saúde Pública do estado da Bahia (LACEN-BA), Salvador, Bahia, Brazil; ^10^Laboratório Central de Santa Catarina (LACEN-SC), Florianópolis, Santa Catarina, Brazil; ^11^Instituto Evandro Chagas, Belém, Pará, Brazil; ^12^Laboratório Central de Saúde Pública do Estado de São Paulo (LACEN-SP), Instituto Adolfo Lutz, São Paulo, São Paulo, Brazil; ^13^Departamento de Imunização e Doenças Transmissíveis (DEIDT)/Secretaria de Vigilância em Saúde (SVS)/Ministério da Saúde (MS), Brasília, Distrito Federal, Brazil

**Keywords:** influenza A virus, resistance, neuraminidase (NA) inhibitors, oseltamivir, baloxavir marboxil, adamantane

## Abstract

The influenza A virus (IAV) is of a major public health concern as it causes annual epidemics and has the potential to cause pandemics. At present, the neuraminidase inhibitors (NAIs) are the most widely used anti-influenza drugs, but, more recently, the drug baloxavir marboxil (BXM), a polymerase inhibitor, has also been licensed in some countries. Mutations in the viral genes that encode the antiviral targets can lead to treatment resistance. Worldwide, a low prevalence of antiviral resistant strains has been reported. Despite that, this situation can change rapidly, and resistant strain surveillance is a priority. Thus, the aim of this was to evaluate Brazilian IAVs antiviral resistance from 2017 to 2019 through the identification of viral mutations associated with reduced inhibition of the drugs and by testing the susceptibility of IAV isolates to oseltamivir (OST), the most widely used NAI drug in the country. Initially, we analyzed 282 influenza A(H1N1)pdm09 and 455 A(H3N2) genetic sequences available on GISAID. The amino acid substitution (AAS) NA:S247N was detected in one A(H1N1)pdm09 strain. We also identified NA:I222V (*n* = 6) and NA:N329K (*n* = 1) in A(H3N2) strains. In addition, we performed a molecular screening for NA:H275Y in 437 A(H1N1)pdm09 samples, by pyrosequencing, which revealed a single virus harboring this mutation. Furthermore, the determination of OST IC_50_ values for 222 A(H1N1)pdm09 and 83 A(H3N2) isolates revealed that all isolates presented a normal susceptibility profile to the drug. Interestingly, we detected one A(H3N2) virus presenting with PA:E119D AAS. Moreover, the majority of the IAV sequences had the M2:S31N adamantanes resistant marker. In conclusion, we show a low prevalence of Brazilian IAV strains with NAI resistance markers, in accordance with what is reported worldwide, indicating that NAIs still remain an option for the treatment of influenza infections in Brazil. However, surveillance of influenza resistance should be strengthened in the country for improving the representativeness of investigated viruses and the robustness of the analysis.

## Introduction

Influenza virus (IV) is an important pathogen that causes respiratory infections and a high disease burden worldwide, often presenting as annual epidemics. The acute respiratory and febrile illness is normally self-limiting in healthy people, but it can be especially threatening in high-risk individuals such as elderly people, children, pregnant women, immunocompromised individuals, and patients with underlying chronic diseases. Per year, influenza infections are reported in 1 billion cases and up to 650,000 deaths worldwide (1). Belonging to the *Orthomyxoviridae* family, IVs are enveloped and contain a segmented genome composed of negative-sense single-stranded RNA (2). Importantly, influenza A virus (IAV) has the potential for causing pandemics, and its strains are classified according to their hemagglutinin (HA) and neuraminidase (NA) surface glycoproteins, with the subtypes A(H1N1)pdm09 and A(H3N2) currently being the most relevant to human health.

Influenza viruses have a well-characterized seasonal circulation pattern in some parts of the world, with peaks during the coldest seasons (autumn-winter). However, Brazil does not have a homogeneous seasonality pattern, due to its continental dimensions, and each country region can have specific patterns for IVs circulation over the year, which is influenced by the different climates present in the country (3). There are three distinct climatic patterns in Brazil, namely, equatorial, in the north and part of the northeast; tropical, in the northeast, midwest, and southeast; and subtropical (temperate), in the south of the country. In view of this, the influenza detection in the country is distinct by comparing the northern equatorial regions and the southern temperate regions. According to the Brazilian Ministry of Health, in 2019, 1,122 fatalities due to IVs infection were reported (4).

To control the high morbidity and mortality caused by IAV infections, two main strategies are currently employed, namely, the annual vaccination for target high-risk populations and the treatment with antiviral drugs. Even though the vaccination against influenza is the most effective form of prevention, it still faces some challenges, such as the absence of a universal influenza vaccine and the need to update vaccine strains included in the vaccines regularly (5). In contrast, antiviral drugs are used for the prophylaxis and control of IAV infections. These compounds act directly on some of the IV's essential proteins, interrupting its replicative cycle and presenting benefits, for instance, shortening the duration of the disease symptoms, and improving clinical outcomes if administered early in the infection (6, 7). Antiviral drugs are also important because they can act as the first line of defense for the population at particular risk in the event of the emergence of a new IAV strain, since the mass production of an effective vaccine can take months. At present, there are several pharmacological classes of drugs with anti-IV activity approved for use. The adamantanes, such as amantadine and rimantadine, act by blocking the viral M2 ion channel (8). Neuraminidase inhibitors (NAIs), represented by oseltamivir (OST), zanamivir (ZAN), peramivir (PER), and laninamivir (LAN), bind to NA and prevent the release of the newly generated virions (9). The drug baloxavir marboxil (BXM), which is a cap-dependent endonuclease inhibitor, targets the polymerase acid (PA) protein, one of the three subunits that constitute the IAV RNA polymerase, inhibiting its cap-snatching mechanism, hence inhibiting mRNA transcription and ending viral replication (10). BXM was initially released for use in 2018 and has been approved in over 30 countries around the world, such as Japan, the USA, and the European Union (11).

However, due to the constant evolution of IAVs, through genetic drift, mutations may arise in the genes that encode viral proteins targeted by antiviral drugs, leading to amino acid substitutions (AAS), which may reduce the drug's effectiveness. This affected the adamantanes, which have not been recommended for the treatment of IAV infections since the 2005–2006 seasons. AASs such as M2:S31N, which remains fixed in IAVs, led to viral resistance to this pharmacological drug class and showed good viral fitness (12). Despite that, in 2017, a cluster of IAV sensitive to the adamantanes was detected in Australia, but their dissemination has not been observed (13).

In relation to NAIs resistance, some AASs in NA correlating with reduced inhibition (RI) to these drugs have been identified. The NA:H275Y was previously detected in high prevalence in the former A(H1N1) viruses that circulated until the end of the 2000s, causing high RI to OST, a fact that impaired the value of this drug (14) at that time. However, the emergence of the pandemic strain A(H1N1)pdm09, which did not have NA:H275Y, allowed the return of OST use to treat those infections. Since then, surveillance of NAIs-resistant strains has been intensified, with the detection of clusters of viruses carrying resistance markers in the USA, Australia, and Japan (15–20). Despite that, further dissemination of these mutated viruses has not been observed. Thus, in the most recent global reports, the frequency of resistant strains has remained low (<1%) (18).

Regarding BXM, which has not yet been approved for use in Brazil, resistance markers in the PA, associated with RI, have been identified during the drug initial trials and in IAVs circulating in some countries, with PA:I38T being the most relevant one (21–24).

Brazilian surveillance of NAIs-resistant IAVs has identified A(H1N1)pdm09 strains carrying the markers NA:H275Y, NA:S247N, and NA:I223K, with a low frequency (25–27). IVs antiviral susceptibility surveillance is conducted by the World Health Organization (WHO) through the Global Influenza Surveillance and Response System (GISRS), which comprises six WHO Collaborating Centers (CCs) and 141 institutions in 111 WHO member states. Brazil is part of the GISRS and has an influenza surveillance system (ISS) with 3 national influenza centers (NICs), which are located at the Respiratory Virus and Measles Laboratory of the Oswaldo Cruz Foundation in Rio de Janeiro (Fiocruz/RJ), the Virology Laboratory of the Evandro Chagas Institute in Pará (IEC/PA), and the Respiratory Virus Laboratory of the Adolfo Lutz Institute in São Paulo (IAL/SP). Brazilian ISS comprises sentinel units for influenza-like illness (ILI) and severe acute respiratory infection (SARI) that are spread across the country collect the samples, which are further sent to the Central State Laboratories (LACENs) located in each of the 27 Brazilian states. Then, a subset of the samples received by the LACENs is sent to the NICs (28).

This study presents the analysis of Brazilian IAVs susceptibility to antivirals in Brazil between 2017 and 2019. We evaluated viral genetic sequences of circulating viruses and performed functional phenotypic analysis by determining the OST concentration that inhibited 50% of NA activity (IC_50_) of Brazilian IAV isolates from the period.

## Materials and methods

### IAV genetic sequences

The Brazilian IAV sequences that were available in the EpiFlu platform in the Global Initiative on Sharing Influenza database (GISAID) (https://www.gisaid.org/) were downloaded and further evaluated. For that, we used the following screening criteria: type: A, host: human, region: South America/Brazil, and collection date: from 1 January 2017 to 31 December 2019. Duplicate sequences were removed. The analysis of the presence of antivirals resistance markers was performed by using the FluSurver tool (https://www.gisaid.org/epiflu-applications/flusurver-app/).

### Samples and data collection

Our laboratory is an NIC for WHO and the Brazilian Ministry of Health and, therefore, continuously receives a subset of samples from ISS collected in 9 of 27 Brazilian states. Respiratory secretion samples, previously characterized as positive for IAV in their respective LACENs, collected from 2017 to 2019, were included in this study. These samples were collected through nasopharyngeal swabs or aspirates from patients displaying ILI or SARI. A case of ILI is defined as the presence of fever, even if reported, accompanied by cough or sore throat and, at least, one of the following symptoms: headache, myalgia, or arthralgia, in the absence of another specific diagnosis. In children aged <2 years, it is defined as the presence of fever (even if reported) and symptoms (cough, coryza, and nasal obstruction), in the absence of another specific diagnosis. SARI cases were defined as cases requiring hospitalization and presenting dyspnea or one of the following signs of severity: peripheral capillary oxygen saturation <95%, respiratory distress, or acute respiratory insufficiency.

### Ethics

This study was approved by the FIOCRUZ—Oswaldo Cruz Institute (IOC) Ethics Committee under the number 68118417.6.0000.5248.

### Pyrosequencing

A molecular screening was performed in influenza A(H1N1)pdm09 positive samples in order to identify the most relevant and frequently detected NAIs resistance marker, the NA:H275Y. We selected representative samples by using the following criteria: epidemiological week, Brazilian State, severe and fatal cases, OST administration, and availability of reagents. For that, the RNA of the clinical samples was extracted using a viral RNA mini kit (QIAGEN, USA), according to the manufacturer's instructions, and then an RT-PCR reaction was performed, followed by a pyrosequencing reaction and analysis using the PyroMark™ Q96 ID Platform. The run was performed in single-nucleotide polymorphism (SNP) mode, and analysis of results was performed by both SNP and allele quantification (AQ) modes, as previously described (29). The NA:H275Y substitution is characterized by the punctual exchange of the guanine (G) nucleotide by the adenine (A) nucleotide, where the GTG triplet would be changed to ATG triplet in the analyzed sequence, which was 7 bases long and is contained into NA catalytic site.

### Cell culture and viral isolation

Virus isolation was performed for all IAV-positive clinical samples received at the laboratory. Madin-Darby canine kidney (MDCK) cells were cultured in Dulbecco's modified Eagle's medium (DMEM™) (Gibco, USA) supplemented with 10% fetal bovine serum (FBS) (Gibco, USA), 100 U/ml penicillin (Gibco, USA), and 100 μg/ml streptomycin (Gibco, USA). After removing the culture medium, 100 μl of the clinical sample was added and incubated at 37°C in 5% CO^2^ in the presence of 2 μg/ml TPCK-treated trypsin (Sigma, USA). Visualization of the cytopathic effect (CPE) for up to 72–96 h post-infection was performed. After observing CPE, the viral isolation was confirmed by using NA activity assay with the chemiluminescent kit NA-STAR (Thermofisher, USA). For the viruses with antivirals resistance markers, we also tried additional passages in cultured cells, even if they do not show any CPE after the first inoculation.

### Functional antiviral assay

Determination of OST IC_50_ (drug concentration to inhibit 50% of NA activity) was done by measuring the NA activity, in the presence of increasing concentrations of OST, by using the chemiluminescent kit NA-STAR™ (Thermofisher™). To this end, NA activity was measured in relative light units (RLUs) on the FLUOstar™ Optima equipment (BMG Labtech™). Classification of the susceptibility profile of the isolates was performed according to the WHO criteria for the OST IC_50_ values for the IAVs, by comparing the IC_50_ median to previously tested viruses (30). Thus, the classification was performed as follows: normal susceptibility, when the IC_50_ value is up to 10 times the median value; RI, when the IC_50_ value corresponds to an increase of 10–100 times in the median value; and high reduced inhibition (HRI), when the IC_50_ value corresponds to an increase in more than 100 times of the median value.

### Statistical analysis

The median IC_50_ of the isolates, 95% confidence interval (95% C.I.), and the analysis of the variance of the medians between isolates, clinical samples, and collection year were calculated using Kruskal–Wallis's test, followed by the Dunn's test for multiple comparisons. All analyses were carried out by using the GraphPad Prism software version 8.0.

## Results

### Identification of NA mutations associated with NAIs RI

We analyzed the sequences of the NA, the gene of interest for NAIs resistance monitoring, from IAVs that circulated in Brazil in the period of 2017–2019 that were available on GISAID (Supplementary Table 1). We evaluated 280 complete NA segments from A(H1N1)pdm09 viruses as well as 450 complete NA segments from A(H3N2) (Table 1). We identified one A(H1N1)pdm09 strain (A/Ceara/152545-IEC/2018) with the NA:S247N AAS collected from a patient who was infected in 2018. In addition, we detected six A(H3N2) strains harboring the NA:I222V AAS (A/Parana/99/2017, A/Parana/152/2017, A/Parana/235/2017, A/Parana/340/2017, A/Brazil/399/2017, and A/Parana/490/2017) and one with the N329K AAS (A/Espirito Santo/174/2017), which were collected in 2017. The clinical and epidemiological data of these cases are summarized in Table 2.

**Table 1:** Brazilian IAVs genetic sequences NA, PA, and M genes available at the EpiFlu database on GISAID by gene and year and amount of AAS found in each year.

**Year**	**A(H1N1)pdm09**			**A(H3N2)**		
	**NA**	**M2**	**PA**	**NA**	**M2**	**PA**
2017	10	7	7	236	111	108
2018	137	115	108	108	89	86
2019	132	86	84	106	75	71
Total	280	208	199	450	275	265

NA, Neuraminidase; AAS, Amino acids substitution, M2, Matrix 2; PA, Polymerase Acid.

**Table 2:** Clinical and epidemiological characteristics of the cases that were infected by the IAVs carrying AAS associated with antivirals RI.

**Virus**	**IAV subtype**	**Gene**	**AAS**	**ID** ** GISAID**	**Brazilian state**	**Gender**	**Age (years)**	**Hospita** **lization**	**SARI**	**Fatal**	**Collection date**	**OST initiation**
A/Espirito Santo/174/2017	A(H3N2)	NA	N329K	EPI_ISL_274162	ES	M	34	No	No	No	03/10/2017	NT
A/Parana/99/2017	A(H3N2)	NA	I222V	EPI_ISL_268344	PR	M	42	No	No	No	02/15/2017	03/07/2017
A/Parana/152/2017	A(H3N2)	NA	I222V	EPI_ISL_268348	PR	M	86	Yes	NI	No	03/07/2017	03/10/2017
A/Parana/235/2017	A(H3N2)	NA	I222V	EPI_ISL_274170	PR	M	50	Yes	Yes	No	04/14/2017	04/14/2017
A/Parana/340/2017	A(H3N2)	NA	I222V	EPI_ISL_274655	PR	M	8	Yes	Yes	No	05/12/2017	05/11/2017
A/Brazil/399/2017	A(H3N2)	NA	I222V	EPI_ISL_275869	SC	M	28	NI	Yes	Yes	21/05/2017	NT
A/Parana/490/2017	A(H3N2)	NA	I222V	EPI_ISL_300393	PR	F	15	No	No	No	06/26/2017	NT
A/Brazil/339/2017	A(H3N2)	PA	E199D	EPI_ISL_275866	PR	M	21	No	No	No	05/02/2017	NT
A/Ceara/152545-IEC/2018	A(H1N1) pdm09	NA	S247N	EPI_ISL_320232	CE	F	3	NI	NI	NI	04/06/2018	NI
A/Espirito Santo/974/2019	A(H1N1) pdm09	NA	H275Y	ND	ES	M	41	No	No	No	09/04/2019	09/04/2019

IAV, Influenza A virus; AAS, Amino Acid Substitution; ID GISAID, Identification on GISAID; SARI, Severe Acute Respiratory Infection; OST, Oseltamivir; ES, Espírito Santo; PR, Paraná; SC, Santa Catarina; CE, Ceará; NA, Neuraminidase; PA, Polymerase Acid; M, Male; F, Female; ND, Not deposited; NI, Not Informed; NT, Not treated.

To complement the surveillance of Brazilian viruses that would pose a threat to the efficiency of the anti-influenza treatment, a pyrosequencing molecular screening was performed specifically to identify the NA:H275Y AAS, as it is the most frequently and importantly detected NAI marker in A(H1N1)pdm09 viruses. In the study period, the laboratory received 1,112 A(H1N1)pdm09 samples, from which 437 were successfully analyzed by this methodology. We identified one strain presenting the NA:H275Y marker (A/Espírito Santo/974/2017), which was collected in September 2019 from a 41-year-old male patient from Espírito Santo State located in the Brazilian Southeastern region. The infected patient presented common ILI symptoms in addition to dyspnea, initiating OST treatment on the same day of sample collection, but did not require hospitalization (Table 2). Allele quantification analysis showed that the sample had 100% of its AA residues at position 275 of NA mutated, which shows the predominance of the mutation in the patient's viral populations. With the aim to verify the spread of H275Y mutated A(H1N1)pdm09 variants in the region where the sample A/Espirito Santo/974/2019 was detected, molecular screening was performed in further 18 samples from the same Brazilian State and period (from August to October 2019). However, no additional samples were detected showing the NA:H275Y AAS.

### Susceptibility of Brazilian IAV isolates to OST

**Figure 1:**
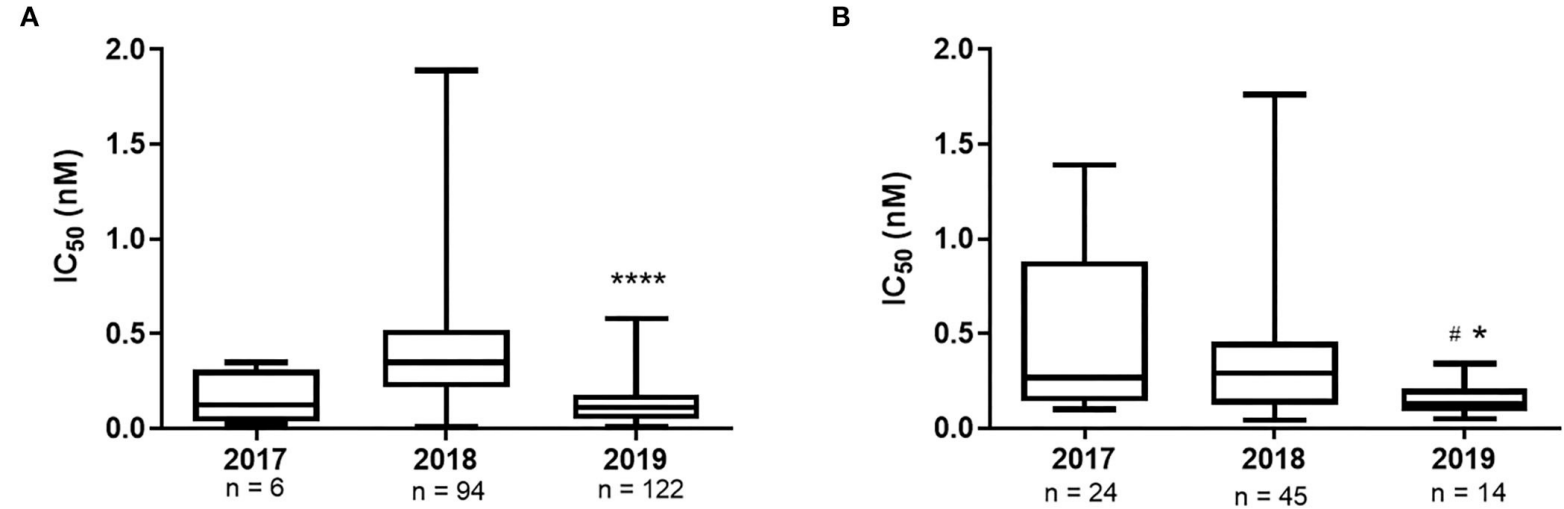
OST IC50 values of Brazilian IAV isolates A(H1N1)pdm09 **(A)** and A(H3N2) **(B)**, by year. ****p value <0.0001 2019 versus 2018, *p value <0.05 2019 versus 2017, ^#^*p* value <0.05 2019 versus 2018. The median variance was obtained using the Kruskal-Wallis test.

The gold standard for the determination of the susceptibility of IAVs to antivirals is through the functional analyses of the isolates in direct contact with the drug to determine OST IC_50_. Over the study period, we successfully isolated 222 A(H1N1)pdm09 and 83 A(H3N2) strains from the 1,987 IAV samples received at the NIC. The A(H1N1)pdm09 isolates the presented IC_50_ values ranging from 0.02 to 0.35 nM in the 2017 (median of 0.13 nM); 0.01 to 1.89 nM in 2018 (median of 0.35 nM); and 0.01 to 0.58 nM in 2019 (median of 0.11 nM). Moreover, the A(H3N2) isolates the presented IC_50_ values ranging from 0.10 to 1.39 nM (median of 0.27 nM) in 2017; 0.04–1.76 nM (median of 0.29 nM) in 2018; and 0.05–0.34 nM (median of 0.13 nM) in 2019 (Table 3). As a result, all the tested isolates showed a normal susceptibility profile to OST, according to WHO criteria (Figure 1). It is noteworthy that none of the viruses that presented any NAI RI mutation were successfully isolated as they did not show any CPE during isolation protocols nor had any NA activity detected after cell culture passages.

**Table 3:** IAVs isolates OST IC_50_ median by year.

**Season**	**A(H1N1)pdm09**			**A(H3N2)**		
	***N***	**Median (nM)**	**95% C.I**.	***N***	**Median (nM)**	**95% C.I**.
2017	6	0.13	(0.02, 0.35)	24	0.27	(0.14, 0.8)
2018	94	0.35	(0.31, 0.43)	45	0.29	(0.16, 0.39)
2019	122	0.11	(0.08, 0.13)	14	0.13	(0.07, 0.23)

CI, Confidence interval.

### Identification of M2 and PA mutations associated with adamantanes and BXM

Further complementing the genetic analysis of the IAVs that circulated in Brazil during the proposed period, we reviewed their M2 and PA gene sequences available in the GISAID database with the objective to identify markers associated with RI to the adamantanes and BXM. A total of 483 M2 gene sequences were evaluated (208 from H1N1pdm09 and 275 from H3N2 viruses) and, as expected, M2:S31N AAS was predominantly found in Brazilian IAV sequences. However, one sample collected from an A(H3N2) strain (A/Brazil/358/2017) had no adamantane resistance marker. This virus was recovered in May 2017 from an 82-year-old female patient who was hospitalized with SARI in Espírito Santo State (Table 2).

In addition, a review of available Brazilian IAV complete PA sequences deposited at GISAID retrieved 464 results (199 from H1N1pdm09 and 265 from H3N2 viruses). Despite not identifying any A(H1N1)pdm09 virus harboring BXM RI-associated mutations, we detected one A(H3N2) PA sequence presenting the PA:E199D AAS (A/Brazil/339/2017) that was collected in May 2017 from a 21-year-old female patient who lived in Paraná State, Brazilian Southern region, and showed mild ILI symptoms (Table 2).

## Discussion

The availability of antiviral treatments to any emerging and reemerging infectious disease, such as caused by IVs, is critical for public health responses and control of the morbidity and mortality of these infections. In Brazil, influenza treatment is based on the NAIs OST and ZAN, of which oral OST is the most widely available drug to treat these infections, followed by the inhaled ZAN (31). BXM treatment, which has already been approved in countries including USA, Europe, and Asia, is still not authorized for use in Brazil (10, 22, 32).

This study evaluated the circulation of IAV strains with genetic markers associated with RI to the antivirals NAIs, adamantanes, and BXM, in the period between 2017 and 2019, which circulated in Brazil. Among the mutations detected in this study, the NA:H275Y is the most frequent and relevant marker associated with RI to NAIs. The sample identified with this substitution showed 100% of its amino acid residues altered. Interestingly, this sample was collected on the same day that the individual started treatment with OST, which allows us to speculate that the emergence of AAS was not due to antiviral pressure. Moreover, allele quantification analysis showed that the sample had 100% of its nucleotides mutated at the NA position of AAS, which would suggest that the individual may have been initially infected with a variant virus that was spreading in the community. Despite that, we analyzed additional A(H1N1)pdm09 samples that were collected by the Brazilian ISI from the same region and period and identified no additional mutated strain. When it is present in A(H1N1)pdm09 viruses, NA:H275Y causes a high decrease in the binding affinity between NAIs and NA, which leads to a significant reduction in the effectiveness of OST and PER drugs (33). It is known that this mutation affects viral fitness and may influence the ability of viral transmission and cell replication in cell culture, as we and others demonstrated (34, 35). Despite that, permissive mutations, such as NA:V241I and NA:N369K, can act to compensate for this loss of fitness and are already incorporated in the circulating IAVs around the world, including Brazil (25). Before the 2009 pandemic, the NA:H275Y was incorporated into the genome of seasonal influenza A(H1N1) strains circulating at that time, leading to their resistance to OST. However, the pandemic strain A(H1N1)pdm09, which did not contain this mutation and was sensitive to OST treatment, emerged, spread, and replaced the previous seasonal A(H1N1) strain. Since then, the frequency of NA:H275Y identification in these viruses has remained low (18). In addition, we have identified an A(H1N1)pdm09 virus that harbored the NA:S247N marker, which exclusively causes a significant RI effect when it is associated with the NA:H275Y mutation, causing a synergistic effect, increasing the reduction in OST and PER susceptibility (36). Even though they were not jointly detected in our sample, NA:S247N should be closely monitored.

In relation to the evaluated A(H3N2) viruses, the AAS NA:I222V, which is associated with the reduction of OST active site hydrophobicity, therefore, decreasing favorable drug interactions (37), was identified in six samples. However, similar to NA:S247N, this AAS has a relevant effect on the NAIs susceptibility strictly when it is associated with NA:E119V in A(H3N2) or NA:H275Y in A(H1N1)pdm09 viruses (38). Notably, sample A/Parana/340/2017, identified as carrying AAS NA:I222V, was collected 1 day after starting OST treatment, which cannot exclude antiviral pressure for the emergence of this mutation. This sample came from an 8-year-old individual with SARI. Furthermore, we show the identification of NA:N329K, already reported to cause RI to OST and ZAN (15, 20).

The functional analysis of OST IC_50_ showed that all Brazilian isolates from the period remained sensitive to the drug. We observed that median OST IC_50_ was reduced in 2019, in comparison with the previous years, for both influenza A subtypes, which would be an indicator of the stabilization of the OST sensitivity of the IAVs and a further support on its use for the treatment of IAV infections. Future investigations will confirm this hypothesis.

Our analyses of the M2 IAV gene sequences showed that most of them had the adamantanes resistance marker M2:S31N, as has globally been reported since the 2005–2006 season (39). For this reason, the adamantane drugs are no longer recommended for the treatment of IAV infections in Brazil and elsewhere for more than a decade. Despite that, one A(H3N2) strain (A/Brazil/358/2017) did not present any known adamantanes resistance markers. This sample was collected from a 2017 case, when A(H3N2) was the predominant IAV subtype circulating (40). Interestingly, in the same year, A(H3N2) clusters without any adamantanes resistance markers were also detected in Australia, despite this, their spread was not observed (13). During the emergence of IAVs with M2:231M AAS, it was suggested that their dissemination was less related to the selective pressure exerted by the use of adamantane drugs. Instead, they would have emerged spontaneously in viral variants and were fixed through their interaction with additional advantageous mutations located in other parts of the viral genome, probably through hitch-hiking effect (41).

Herein, we also revealed that BXM susceptibility AASs in PA were detected in a unique A(H3N2) virus that contained PA:E199D. This substitution is in the same position of a distinct one (PA:E199G) that has been reported in association with a discrete (4.5-fold) RI to BXM (22). The position 199 of PA is important for this drug interaction. Nonetheless, further studies need to be performed to confirm whether PA:E199D AAS also affects the effectiveness of BXM. It is worth mentioning that this marker was detected 1 year before the first approval of BXM in the world and until now, no PAI has been approved for use in Brazil, suggesting that this AAS has emerged spontaneously in the PA gene. PA is one of the 3 subunits that make up the IAV polymerase, playing a crucial role in the replication cycle of the virus. The gene encoding the PA protein is highly conserved and the emergence of mutations is a rare event. However, some markers of RI to BXM have already been identified, especially at position PA:I38X (T/F/M/S/L/V), the most frequently found (18).

The monitoring of IAVs to detect reduced susceptibility to antivirals is an essential activity of the surveillance networks for providing information regarding the continuous use of these compounds. However, this study presents some limitations, such as the number of available virus sequences and isolates, which limited a robust assessment of the prevalence of the mutated viruses. In addition, there is a need for a closer monitoring of individuals in the country who are receiving treatment with these antivirals, especially among the immunocompromised groups. Therefore, there is a need to strengthen the Brazilian ISS to address these limitations. Moreover, there is less consistency in the quantity of data and numbers of samples that are collected and evaluated from distinct Brazilian regions, making it important to increase the representativeness of strain identification in some states to strengthen the planning for national vaccination campaigns.

## Conclusion

This study, covering IAVs that circulated in Brazil in the period of 2017–2019, reveals a low prevalence of IAVs with genetic markers associated with resistance to the anti-influenza drugs NAIs and BXM. Our data further demonstrate that the available IAVs isolates from Brazil were sensitive to OST. In addition, the majority of IAVs from the country have the adamantanes resistance markers. Therefore, NAIs remain an option for the control of influenza infections in the country. Monitoring the susceptibility of the viruses to the available treatments is crucial for the guidance of the medical interventions.

## Data availability statement

The raw data supporting the conclusions of this article will be made available by the authors, without undue reservation.

## Ethics statement

This study was approved by the FIOCRUZ—Oswaldo Cruz Institute (IOC) Ethics Committee under the number 68118417.6.0000.5248. Written informed consent to participate in this study was provided by the participants' legal guardian/next of kin.

## Author contributions

TS, MS, and AM: conceptualization. TS, JM, CG, MM, PR, CS, MD, RR, AC, TG, FI, FP, SF, JF, KS, FM, MS, and AM: methodology, analysis, and investigation. MS, AM, and WA: resources. TS, DB, MS, and AM: writing. All authors contributed to the article and approved the submitted version.

## Funding

This project was supported by the Coordenação de Aperfeiçoamento de Pessoal de Nível Superior (CAPES) for providing a doctoral grant for JSCCM and a master grant for TCS; Programa Estratégico de Apoio à Pesquisa em Saúde (PAPES), Fundação Oswaldo Cruz, CNPq, and Coordenação Geral de Laboratórios de Saúde Pública (CGLAB) from the Brazilian Ministry of Health.

## Conflict of interest

The authors declare that the research was conducted in the absence of any commercial or financial relationships that could be construed as a potential conflict of interest.

## Publisher's note

All claims expressed in this article are solely those of the authors and do not necessarily represent those of their affiliated organizations, or those of the publisher, the editors and the reviewers. Any product that may be evaluated in this article, or claim that may be made by its manufacturer, is not guaranteed or endorsed by the publisher.

## Supplementary material

Supplementary Table 1 Brazilian IAVs gene sequences data that were included in this study from GISAID EpiFlu platform.
